# Free Radical Scavenging Capacity, Carotenoid Content, and NMR Characterization of* Blighia sapida* Aril Oil

**DOI:** 10.1155/2018/1762342

**Published:** 2018-08-13

**Authors:** Andrea Goldson Barnaby, Jesse Clarke, Dane Warren, Kailesha Duffus

**Affiliations:** ^1^The Department of Chemistry, The University of the West Indies, Mona, Kingston 7, Jamaica; ^2^College of Health Sciences, Medical Technology Department, University of Technology, Kingston 7, Jamaica

## Abstract

*Blighia sapida* aril oil is rich in monounsaturated fatty acids but is however currently not utilized industrially. The oil was characterized utilizing nuclear magnetic resonance (NMR) and Fourier Transform Infrared Spectroscopy (FTIR). A spectrophotometric assay was conducted to determine the free radical scavenging properties and carotenoid content of the oil. Chemical shifts resonating between *δ* 5.30 and 5.32 in the ^1^H NMR are indicative of olefinic protons present in ackee aril oil which are due to the presence of oleic acid. A peak at 3006 cm^−1^ in the FTIR spectra confirms the high levels of monounsaturation. The oil has a free radical scavenging activity of 48%  ± 2.8% and carotenoid content of 21 ± 0.2 ppm.

## 1. Introduction

**Figure 1:**
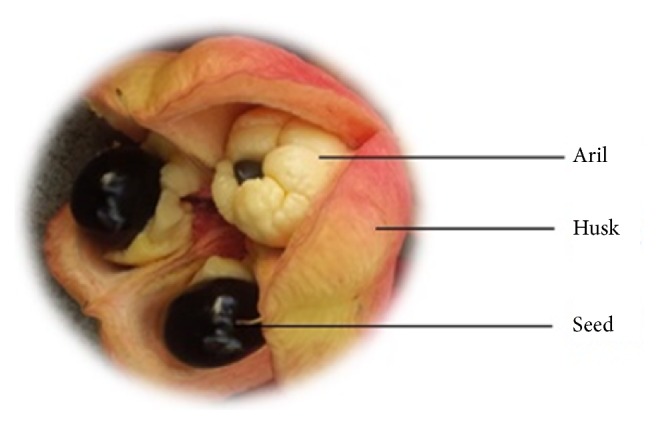
The ackee fruit

*Blighia sapida* Koenig (ackee) (Figure 1) is native to West Africa. The immature fruit is toxic and should not be consumed due to the presence of a nonproteinogenic amino acid, hypoglycin A [1]. Mature arilli is however safe for consumption. In Jamaica, ackee is widely consumed without any ill effects. This is due to the proper harvesting and preparation of the fruit for consumption. The arilli is a major component of the national dish, ackee, and cod fish. As the fruit matures, hypoglycin A is translocated to the seeds where it is converted to hypoglycin B [2]. The concentration of hypoglycin A decreases from approximately 8000 mg/kg in the immature arilli to 271 mg/kg in the mature fruit. The concentration of hypoglycin B in the seeds increases from 1629 to 11774 mg/kg [2]. Immature arilli and the seeds of the fruit should therefore not be eaten. The mode of toxicity of hypoglycin is its metabolism to methylenecyclopropyl-acetyl-CoA which inhibits fat oxidation [1]. Regrettably there are still reports of people falling ill from the consumption of immature ackees. In Nigeria, there were reports of children becoming ill after the consumption of roasted ackee seeds and arilli of the fruit [3]. Children who are unaware of the dangers of the fruit have been more prone to ackee intoxication. Proper education of the potential toxicity of the fruit is therefore necessary. The ackee is not the only source of hypoglycin. Hypoglycin has also been isolated from sycamore maple tree (*Acer pseudoplatanus*) and has been implicated in atypical myopathy observed in horses [4].

The ackee is canned in brine for export to the United States of America, Canada, and Europe. A significant amount of ackee arilli waste is generated from the canning process which may be considered for use in the production of ackee aril oil. Mature ackee arilli contains over 50% lipid and is high in oleic acid [5]. The toxic components of the fruit are water soluble and therefore would not be present in lipid extracts of the fruit. Lipids play an important role in food and nutrition serving as a source of energy, vitamins, and antioxidants [6]. Carotenoids and vitamin E in the form of tocopherols or tocotrienol are examples of natural antioxidants found in plant oils [7]. Antioxidants serve a protective role by scavenging free radicals such as reactive oxygen species that can lead to the damage of cell membranes [8]. Carotenoids are also of commercial significance due to their application as food colorants [9].

In the present study ackee aril oil was characterized utilizing nuclear magnetic resonance and infrared spectroscopy. The free radical scavenging capacity and carotenoid content of ackee aril oil were also evaluated.

## 2. Materials and Methods

### 2.1. Samples

Ackee arilli were obtained from a local processor of canned ackee in brine. Arilli were dried to constant weight (75°C for 1 day, Gallenkamp Laboratory Oven OV-330, England). A composite sample of the dried arilli was extracted with hexane (26°C, 24 h). The resulting extract was concentrated* in vacuo*. Samples of commercial coconut oil and soybean oil were also evaluated to provide a comparison.

### 2.2. Acid Value, Free Fatty Acid, pH, and °Brix

The pH of the oil samples was measured using a pH meter (Oakton, pH Tutor). The acid value was determined by titration of aril oil (10 mL) with sodium hydroxide (0.1 M) utilizing phenolphthalein as indicator [10]. The percentage free fatty acid was expressed based on oleic acid, the predominant fatty acid present in ackee aril oil [5]. The °Brix (total soluble solids) of the oils was determined utilizing a HI96801 refractometer (Hanna instrument, 0-85% Brix, and 26.4°C).

### 2.3. The 1,1-Diphenyl-2-Picrylhydrazyl (DPPH) Radical Scavenging Assay

The DPPH assay was performed according to the method of Brand-Williams [11]. To each oil sample (200 mg), ethanol (2 mL, 80%) containing hydrochloric acid (1%) was added and the resulting mixture reacted with the stable DPPH radical. The reaction mixture consisted of ackee aril oil (0.5 mL), absolute ethanol (3 mL), and DPPH (0.5 mM, 0.3 mL). The reaction was allowed to proceed for 100 min after which the absorbance was measured at 517 nm using a spectrophotometer (Thermo Scientific, Genesys 10S). A mixture of ethanol (3.3 mL) and oil (0.5 mL) served as the blank. A control solution was prepared by mixing ethanol (3.5 mL) with the DPPH radical solution (0.3 mL). The data obtained was used to calculate the free radical scavenging capacity.

(1) $$\begin{array}{c} \%=\left[\;1-\frac{A_{1}}{A_{0}}\;\right]*100 \end{array}$$

where

  A_1_ = absorbance of sample at 517 nm
  A_0_ = absorbance of control at 517 nm

### 2.4. Carotenoid Determination

A spectrophotometric assay was used to determine the carotenoid content of oil samples [7]. Samples (0.5 g) were weighed and transferred to a volumetric flask and n-hexane added (25 mL). The absorbance of the solution was measured at 446 nm.

(2) $$\begin{array}{c} Carotenoid\;\;content\;\left(\;ppm\;\right)=\frac{\left[V\times 383\times \left(A_{s}-A_{b}\right)\right]}{\left(100\times W\right)} \end{array}$$

where

  V = volume used for analysis
  383 = vxtinction coefficient for carotenoids
 
A_s_ = sample absorbance
 
A_b_ = blank absorbance
  W = sample weight (g)

### 2.5. ^1^*H* NMR and ^13^*C* NMR Characterization

^1^H NMR and ^13^C NMR characterization of the oil was performed on a Bruker BioSpin 200 MHz at 200 MHz. Lipid extracts (20 mg) were analyzed in deuterated chloroform (CDCl_3_) at 25°C, with tetramethylsilane (TMS) as the internal standard. The chemical shifts reported is in units of parts per million (ppm). A chemical shift of 1 ppm implies that the magnetic field required to produce the signal is 1 millionth less than that required for TMS.

### 2.6. Fourier Transform Infrared Spectroscopy

A Bruker Vector 22 Fourier Transform Infrared (FTIR) Spectrometer was utilized to record the infrared spectra of ackee oil samples. The FTIR spectrum was recorded between 4000 and 500 cm^−1^. The spectrum was obtained by averaging 20 scans recorded at a resolution of 2 cm^−1^. Spectra were baseline-corrected. OPUS software was used to acquire and manipulate the spectral data.

### 2.7. Data Analysis

Samples were analyzed in duplicate. The mean of the data and the standard error is reported.

## 3. Results and Discussion

### 3.1. Acid Value, Free Fatty Acid, pH, and °Brix

The acid value is a frequently investigated parameter in the edible oil industry. It is a measure of the free fatty acid content of the oil and is an indicator of oil quality [12]. In the presence of the enzyme lipase, triglycerides undergo enzymatic hydrolysis to produce free fatty acids [13]. Free fatty acids are highly susceptible to oxidative rancidity leading to off flavor and odors. High acid values indicate oil degradation and inadequate processing or storage of oils. The acid value of ackee aril oil was 1.3% which is within the required limits observed for oils such as coconut oil < 6 and soybean oil < 2.5 [14]. The oil had a pH of 4.5 (Table 1).

The degree Brix (°Brix) is widely utilized in the food and beverage industries for quality control. It represents the percentage sucrose or dissolved solids in a particular liquid. Of the oils investigated, the highest °Brix was observed in soybean oil (73) followed by ackee oil (69) and coconut oil (66). Palm oil and olive oil have been reported as having a °Brix in the range of 70–75 [15].

### 3.2. Free Radical Scavenging Activity

Free radical scavenging activity observed in oils is due to the presence of fat soluble antioxidants. Tocopherols are responsible for the antioxidant activity of nut oils. In a study conducted by Arranz [16] pistachio nuts were found to have the greatest antioxidant capacity. Unrefined ackee oil exhibited a free radical scavenging activity of 48 ± 2.8%. The radical scavenging properties of the oil may be due to the presence of carotenoids and phenolic compounds. The major phenolic compounds present in olive oil are oleuropein, hydroxytyrosol, and tyrosol [17]. Vegetable rich diets, such as the Mediterranean diet, are believed to contribute to a reduction in coronary heart disease, prostate, and colon cancers [17]. Coconut oil had a free radical scavenging activity of 28 ± 7.1% and soybean oil of 100%. The high free radical scavenging activity of refined soybean oil is expected due to the addition of antioxidants to the refined product.

### 3.3. Carotenoid Content

Carotenoids are derivatives of terpene and are also referred to as tetraterpenoids. They are lipid soluble and contribute to the yellow and orange colour of fruits and vegetables. Some vegetable oils also contain carotenoids. The actual concentration depends on the source. Palm oil is a good source of carotenoids containing between 500 and 700 ppm [18]. The major carotenoids in palm oil are *α*- and *β*-carotenes. In a study conducted by Dauqan [7] red palm oil contained the highest levels of *β*-carotene (542 ppm). Ackee aril oil was found to contain low concentrations of *β*-carotene (21 ± 0.2 ppm). Carotenoids are believed to impart health benefits by reducing the incidence of certain cancers and eye disease [19]. The beneficial effects of carotenoids are partly due to their antioxidant properties. Coconut oil (0.34 ± 0.1 ppm) and soybean oil (1.39 ± 0.1 ppm) were a negligible source of carotenoids.

### 3.4. Nuclear Magnetic Resonance (NMR) Spectroscopy

**Figure 2:**
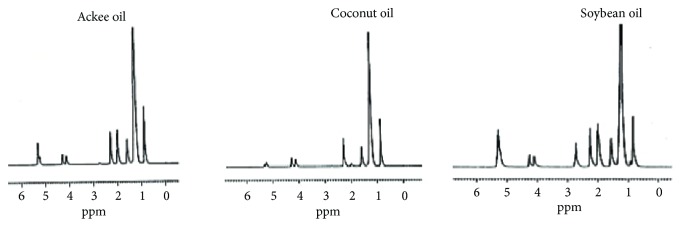
^1^H NMR spectra of ackee, coconut, and soybean oils.

Fatty acids exist primarily as triacylglycerols in oils of plant origin. They are formed predominantly from the unsaturated fatty acids, oleic acid, linoleic acid, and *α*-linolenic acid. Ackee aril oil and soybean oil consist primarily of the unsaturated fatty acids oleic acid (C18:1) and linoleic acid (C18:2), respectively, whereas in coconut oil, lauric acid (C12), a saturated fatty acid, is the predominant fatty acid [5, 20, 21]. NMR spectroscopy has been utilized in the characterization of lipids and to detect adulteration [22, 23]. It may also be utilized as an indicator of the level of unsaturation present in the oils being investigated. In the ^1^H NMR data the terminal methyl protons from the acyl side chains of the triacylglycerols were observed at *δ* 0.84 while the methylene protons resonated at *δ* 1.58 (Figure 2, Table 2). The most significant difference in the ^1^H NMR spectral data was within the region of *δ* 1.98 and *δ* 2.73 (Figure 2, Table 2). Protons attached to the bis-allylic carbon (CH=CH-C**H**_**2**_-CH=CH) in linoleic acid (*δ* 2.73) were most pronounced in soybean oil with only a very small peak being observed in ackee aril and coconut oil samples indicating that linoleic acid is present in minimal quantities in ackee aril oil and coconut oil. A singlet at *δ* 1.98 is due to the methylene protons (C**H**_**2**_-CH=CH) adjacent to the methine carbon in unsaturated fatty acids [24]. This peak was most pronounced in ackee aril and soybean oils which contains monounsaturated and polyunsaturated fatty acids, respectively, but it is least evident in coconut oil which consists primarily of saturated fatty acids [21]. Olefinic protons resonated between *δ* 5.30 - 5.32 (C**H**=C**H**). The ^1^H NMR also revealed the presence of two doublet of doublets (*δ* 4.11-*δ* 4.13; *δ* 4.27-*δ* 4.28) and a multiplet (*δ* 5.24) (Table 2) which is characteristic of the protons on the glyceryl moiety of triacylglycerols.

**Figure 3:**
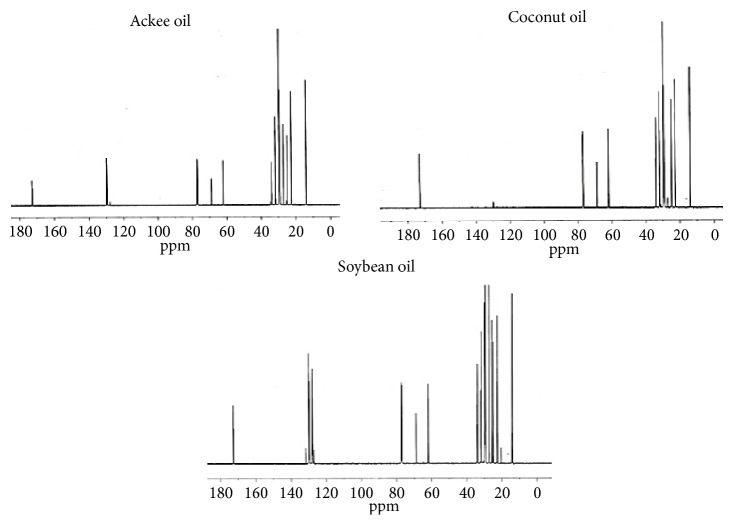
^13^C NMR spectra of ackee, coconut, and soybean oils.

The ^13^C NMR of the oils investigated showed evidence of the presence of both oleic acid and linoleic acid which are present in varying percentages in all the oils investigated [5, 25, 26]. The carbon double bonds present in oleic acid and linoleic acid typically resonate between *δ* 127 and *δ* 129, respectively [24]. Of the oils investigated only soybean contains linolenic acid which has three centers of unsaturation [26]. These carbons resonated in the region of *δ* 128.01-128.38 which was a characteristic feature of the ^13^C NMR spectrum of soybean oil compared to the spectral data of ackee aril oil and coconut oil (Figure 3 and Table 3). Peaks observed between *δ* 14 and *δ* 29 are due to the acyl side chains of triacylglycerols [24]. The *α* and *β* carbons on the triacylglycerol backbone resonated at *δ* 62 (*α*-CH_2_O), *δ* 69 (*β*-CH_2_O), *δ* 172 (*α*-C1), and *δ* 173 (*β*-C1).

### 3.5. Fourier Transform Infrared Spectroscopy (FTIR)

**Figure 4:**
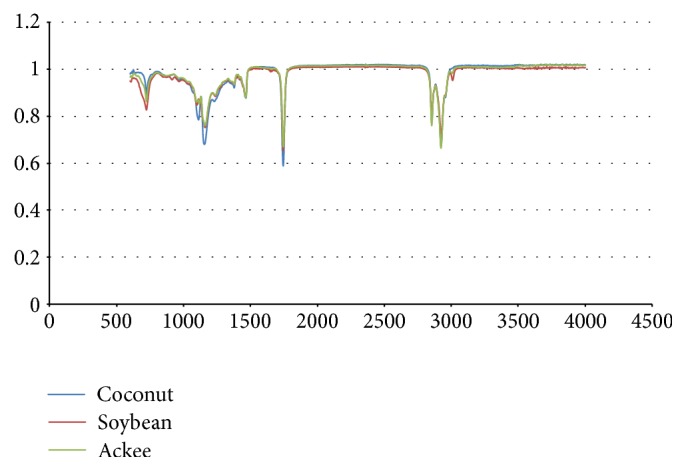
FTIR spectra of ackee, coconut, and soybean oils.

FTIR may be utilized in sample authentication, detection of adulteration, and identification of the major functional groups that are present in the samples analyzed [27–29]. Significant absorption bands were observed at 2922 cm^−1^, 2852 cm^−1^, and 1743 cm^−1^ in the infrared spectra of the oils investigated (Figure 4, Table 4). Peaks at 2922 cm^−1^ and 2852 cm^−1^ are attributable to aliphatic stretching vibrations of the acyl side chain in triacylglycerols [30]. The sharp band at 1743 cm^−1^ is due to carbonyl stretching of carboxyl ester functionalities [31]. Olefinic carbons (HC=CH) were observed between 719 cm^−1^ and 721 cm^−1^. A shoulder peak observed at 3006 cm^−1^ is indicative of the presence of unsaturated fatty acids, specifically the olefinic group =CH [32]. This peak was noticeably absent from the spectral data of coconut oil which is a saturated oil.

## 4. Conclusions

Lipids play an important role in diet and health. A comparative analysis was made of the free radical scavenging activity, carotenoid content and NMR profile of ackee aril oil, coconut oil, and soybean oil. Of the oils investigated, ackee aril oil had the highest carotenoid content. Its free radical scavenging property was intermediate to that of coconut oil and soybean oil. Several characteristic peaks were observed in the NMR and FTIR spectral data which confirms that oleic acid is the major fatty acid present in ackee aril oil. Ackee aril oil may be considered for commercial food applications.

## Figures and Tables

**Table 1:** Physicochemical properties of ackee, coconut, and soybean oils.

**Parameter**	**Coconut**	**Ackee**	**Soybean**
Brix	66°	69°	73°
pH	3.6	4.5	5.2
Acid value	0.6	1.3	0.03
Free fatty acid (%)	0.2	0.1	0.1
Carotenoid (ppm)	0.34 ± 0.1	21.0 ± 0.2	1.39 ± 0.1
Free radical scavenging activity (%)	28 ± 7.1	48 ± 2.8	100

**Table 2:** ^1^H Nuclear magnetic resonance spectroscopy of ackee, coconut, and soybean oils.

**Proton**	**Functionality**	**Ackee oil**	**Coconut**	**Soybean**
		**δ** ** ppm**	**δ** ** ppm**	**δ** ** ppm**
C**H**_**3**_	Terminal methyl	0.84 (t)	0.84 (t)	0.84 (t)
C**H**_**2**_	Methylene	1.24 (s)	1.24(s)	1.23 (s)
C**H**_**2**_-CH_2_-COO	Acyl chains	1.58 (s)	1.59 (s)	1.57 (m)
C**H**_**2**_-CH=CH	All unsaturated fatty acids	1.98 (s)	*∗*1.99 (s)	2.02 (m)
C**H**_**2**_-COO	All acyl chains	2.29 (m)	2.29 (m)	2.28 (m)
C=C-C**H**_**2**_=C	Protons attached to bis-allylic carbon	*∗*2.73 (s)	*∗*2.73 (s)	2.73 (m)
C**H**_**2**_O(*α*)	Glycerol (triglycerides)	4.13 (dd)	4.12 (dd)	4.11 (dd)
		4.28 (dd)	4.29 (dd)	4.27 (dd)
C**H**O(*β*)	Glycerol (triglycerides)	5.24 (m)	5.24 (m)	5.24 (m)
C**H**=C**H**	Olefinic protons	5.30 (m)	5.31 (m)	5.32 (m)

m: multiplet; s: singlet; dd: doublet of doublet; t: triplet.

*∗*Very small peak.

**Table 3:** ^13^C Nuclear magnetic resonance spectroscopy of lipid extracts.

**Carbon**	**Assignment**	**Ackee oil**	**Coconut oil**	**Soybean oil**
		**δ** ** (ppm)**	**δ** ** (ppm)**	**δ** ** (ppm)**
*α*-CH_3_	Acyl chains	14.02	14.08	14.06
*β*-CH_3_	Acyl chains	22.62	22.57; 22.65	21.0, 22.62
C3	Acyl chains	25.57; 26.87	24.49, 24.82,	25.57
			24.86, 25.58	
C8–11 (oleyl)	Allylic	27.12	27.12	27.13
C8–14 (linoleyl)		27.17	27.17	27.14
CH_2n_	Acyl chains	29.01-29.74	28.89-29.73	29.00-29.74
C16	Linoleyl	31.50, 31.58	31.21, 31.49,	30.34, 30.76
		31.78, 31.90,	31.63. 31.84,	31.49, 31.77
		31.92	31.89	31.90, 31.91
*α*- C2	Acyl chains	33.91, 33.93	33.97	32.52, 33.89
*β*-C2	Acyl chains	34.07, 34.48,	34.14	34.05
		34.62		
*α*-CH_2_O	Glycerol moiety	61.98	62.02	61.97, 64.90
*β*-CH_2_O	Glycerol moiety	68.88	68.85	68.87
*β*-C9	Oleyl	127.83, 128.01	127.84, 129.62	127.06-127.99
*β*-C10	Oleyl	129.54, 129.57	129.90, 130.07	128.01-128.38
		129.68,		129.54-129.97
		129.85,129.99		131.70
*α*-C1	Glycerol moiety	172.52	172.70	172.46
*β*-C1	Glycerol moiety	172.91, 172.93	173.10	172.83, 172.86

**Table 4:** Infrared spectra data for ackee, coconut, and soybean oil.

**Functionality**	**Ackee oil**	**Coconut oil**	**Soybean oil**
	**c** **m** ^−1^	**c** **m** ^−1^	**c** **m** ^−1^
Olefinic carbons –HC=CH-cis	721 (m)	721 (m)	719 (m)
C-O-C stretching in esters		1078 (w)	1031; 1097 (w)
C-O-C stretching in esters	1161 (m)	1155 (s)	1159 (s)
C-O-C stretching in esters	1234 (w)	1232 (w)	1242 (w)
CH_3_ bending	1379 (w)	1377 (m)	1379 (w)
CH_3_ deformation and/or C-H bending mode of CH_2_		1435 (w)	
CH_2_ bending (acyl chain) and/or CH_3_ deformation	1464 (m)	1464 (m)	1462 (m)
C = O stretching in esters	1743 (s)	1743 (s)	1743 (s)
C-H stretching in CH_3_ and CH_2_ (*ν*_sym_)	2852 (s)	2852 (s)	2854 (m)
C-H stretching in CH_3_ and CH_2_ (*ν*_antisym_)	2922 (s)	2922 (s)	2924 (s)
C-H stretching in CH_3_ (*ν*_asym_) and CH_2_	2954 (sh)	2956 (sh)	2956 (sh)
C-H stretching vibration, olefinic group =CH	3006 (sh)		3006 (sh)

Abbreviations† s: sharp; m: medium; w: weak; sh: shoulder.

## Data Availability

The data used to support the findings of this study are included within the article.
